# A Survey of Diagnostic Delay in Axial Spondyloarthritis Across Two National Health Service (NHS) Rheumatology Services

**DOI:** 10.7759/cureus.23670

**Published:** 2022-03-30

**Authors:** William J Gregory, Jasmin Kaur, Sian Bamford, Hasan Tahir

**Affiliations:** 1 Faculty of Health, Psychology and Social Care, Manchester Metropolitan University, Manchester, GBR; 2 Rheumatology, Northern Care Alliance National Health Service (NHS) Foundation Trust (Salford Care Organisation), Salford, GBR; 3 Rheumatology, Barnet Hospital, Barnet, GBR; 4 Physiotherapy, Royal Free Hospital, London, GBR; 5 Division of Medicne, University College London, London, GBR

**Keywords:** musculoskeletal screening, physiotherapy assessment, survey research, delay to diagnosis, axial spondyloarthritis

## Abstract

Introduction

Diagnostic delay is an ongoing challenge in axial spondyloarthritis (axial SpA). A recent, comprehensive literature review has found a mean average of 8.7 years of delay between symptom onset and formal diagnosis in the United Kingdom (UK). The primary aim of this study was to identify delays to diagnosis experienced by patients with axial SpA under the ongoing care of two urban National Health Service (NHS) rheumatology services. The secondary aims were (a) to count healthcare professional (HCP) interactions after symptom onset but prior to the diagnosis, (b) to compare our data to published delay to diagnosis research and (c) to explore contributing factors locally and the variation between the two UK rheumatology services.

Methods

A 14-question survey was created to identify the delay to diagnosis and contributing factors across two urban NHS axial SpA services, from the onset of symptoms to diagnosis and commencement of treatment. Participants were recruited from clinic visits between August and November 2021 and completed the survey either on paper or via online survey software, both with HCP support.

Results

Those completing the survey formed a cohort of 106 participants with an established diagnosis of axial SpA who attended the axial SpA services at either Royal Free NHS Foundation Trust or Salford Care Organisation, Northern Care Alliance NHS Foundation Trust. The mean time from the onset of symptoms to the diagnosis of axial SpA was similar across centres despite the differences in demographics, with Royal Free at 5.72 years and Salford Royal at 5.96 years. When reviewing via median diagnostic delay, there was a notable difference with Royal Free at 6.09 years and Salford Royal at 4.27 years.

Across the two sites, between the onset of symptoms and the diagnosis of axial SpA, 90% of the participants saw a general practitioner (GP), of which 63% of the patients saw a GP 1-5 times, 23% saw 5-10 times and 14% saw more than 10 times. Many participants also saw other HCPs, including physiotherapists, other manual therapists and hospital specialists prior to diagnosis. In addition, 32% saw one other HCP, 18% two other HCPs, 9% three, 7% four and 2.7% five other HCPs prior to diagnosis. Close to 80% of the patients stated that they had received adequate axial SpA education at diagnosis, and 76% of the patients were aware of who to contact in the event of a flare.

Conclusions

These data highlight that the mean average time to diagnosis for both trusts was between five and six years, somewhat lower than the 8.7-year national UK average. However, despite being specialist centres, these data are a long way from the National Axial Spondyloarthritis Society (NASS) “Gold Standard” of one year time to diagnosis. The contributors to this include lack of HCP and community awareness about axial SpA, its recognition and appropriate onwards referral. There is a need for concerted ways of working for the development of patient pathways and public and HCP education to reduce this delay to allow the ambitious NASS Gold Standard of one year time to diagnosis to be achieved.

## Introduction

Axial spondyloarthritis (axial SpA) is a chronic immune-mediated rheumatic disease with a number of diverse characteristics but classically involves inflammation and new bone formation - predominantly in the axial skeleton. In addition to back pain, people living with axial SpA often experience fatigue, early morning stiffness, sleep disturbance and reduced function and/or mobility [1]. Extra-articular manifestations (EAMs) of axial SpA are often present and can be a pathway to diagnosis; these EAMs include uveitis, enthesitis, psoriasis, dactylitis and inflammatory bowel disease (IBD) [2].

In the United Kingdom (UK), it has recently been shown to take an average of nearly nine years from initial symptom onset for someone living with axial SpA to receive a diagnosis [2]. The reasons for this delay are multi-factorial, and not all may be resolvable; however, aiming to reduce this delay to diagnosis is crucial as any delay can leave those awaiting diagnosis prone to experiencing significant amounts of pain and functional limitation [3,4]. Axial SpA most often begins in early adulthood [5] and hence can impact the life stage where individuals are trying to establish careers and start relationships and families. It has been shown that people living with axial SpA who have had a delayed diagnosis tend to experience a range of poorer outcomes, including decreased spinal mobility, increased radiographic disease progression, poorer quality of life, greater likelihood of work disability, low mood/depression and higher direct and indirect healthcare costs [6].

Previous research has found instances of missed opportunities for earlier identification of a requirement for a referral to rheumatology: this has been seen across consults with general practitioners (GPs) [7] and physiotherapists [8]. In addition to these two groups of healthcare professionals (HCP), it has been suggested that other community-based HCPs may provide consultations where an onwards referral to diagnosis, usually via the GP, could have been made earlier. Furthermore, the non-musculoskeletal EAMs associated with axial SpA could lead to initial suspicions of the diagnosis arising during a consult with ophthalmology, gastroenterology, dermatology or other secondary care-based specialisms. Finally, the potential misdiagnosis of early axial SpA as fibromyalgia [9] means those living with undiagnosed axial SpA may present to specialist pain management services. Recently published Europe-wide data that surveyed over 2500 people living with axial SpA found that the number of HCPs seen before diagnosis significantly correlates with a longer delay to diagnosis [10].

Aims

Our primary aim was to identify the duration of the delay to diagnosis experienced by patients with axial SpA under the ongoing care of two urban NHS axial SpA services. The secondary aims were (a) to count HCP interactions after symptom onset but prior to the diagnosis, (b) to compare our data to published delay to diagnosis research and (c) to explore contributing factors locally and the variation between the two rheumatology services.

## Materials and methods

A 14-question survey was created to identify the duration of delay in diagnosis and contributing factors across two urban National Health Service (NHS) rheumatology services, from symptom onset to diagnosis and treatment commencement. This survey was created by rheumatology staff at the two centres involved and included consultation with those with lived experience of axial SpA to ensure the content and style of the survey were appropriate for self-completion and covered the issues most commonly experienced in diagnostic delay by those living with axial SpA. With this being a multi-site survey, there was an anticipation that the delivery of the survey and the collection of the data would be locally relevant and fit in with survey collection processes available at each site. For this survey-based observational research, ethical approval was not required.

Sample

Participants from clinics within the axial SpA service at Royal Free NHS Foundation Trust and Salford Care Organisation, Northern Care Alliance NHS Foundation Trust were invited to participate. The recruitment of potential participants was from consecutive attendees at the axial SpA clinics at each site over a period of 6-8 weeks. Following verbal, informed consent, with an explanation of the aims of the survey and reassurance about the maintenance of confidentiality through the process, participants were provided with the survey on the day of their consultation to complete on paper, electronically or over the phone. Data from the Royal Free cohort was primarily collected via an in-house online survey portal, with a minority collected via a paper copy of the survey. Data from the Salford Care Organisation cohort was collected via a paper printout of the survey, supplemented by discussions with the HCP in the axial SpA clinic and reference to the electronic health record that covers historic secondary and primary care consults.

Survey content

Questions included demographics and the time to diagnosis along the patient pathway from the first experience of symptoms, first consult with the general practitioner, first appointment in secondary care rheumatology and provision of a definitive diagnosis of axial SpA. To identify if participants were seeing multiple HCPs, including other secondary care-based specialisms, it was also asked which HCPs they saw prior to their axial SpA diagnosis and on how many occasions for each HCP (Supplementary Material 1 in Appendices).

Prior to its launch, the survey was approximated to take 5-10 minutes to complete.

## Results

A cohort of 106 participants with an established diagnosis of axial SpA who attended the axial SpA service at either Royal Free NHS Foundation Trust or Salford Care Organisation, Northern Care Alliance NHS Foundation Trust responded between August and November 2021. Baseline demographics are presented in Table 1.

**Table 1:** Demographics of the participants BASDAI: Bath Ankylosing Spondylitis Disease Activity Index

Measure	Royal Free	Salford Royal
Responses (N)	60	46
Age on undertaking the survey, years (mean)	44	42
Sex, male (%)	55%	72%
BASDAI score, on the day of completing the survey (range)	1-9	0.4-9
On biologics, yes (%)	51.7%	66.6%
Primary language - English (%)	75%	98%
Primary language - other (%)	25% (French, Romanian, Bulgarian, Kurdish, Turkish, Hungarian, Pashto, Tamil and Portuguese)	2% (one person) (Polish)
Ethnicity - White British (%)	56.7%	93.5%
Ethnicity - other (%)	43.3% (Romanian, Turkish, Ashkenazi Jewish, Pashtun, Japanese, Brazilian, Cypriot, Asian, African and Caribbean)	6.5% (Japanese, Jewish and Polish)
Majority area (%)	68.3% (Barnet, Enfield and Hertfordshire)	60% (Salford)
Area - other (%)	31.6% (Greenwich, Harringay, Bedfordshire, Ruislip, Camden, Redbridge, Harrow, Chingford, Surrey and Buckinghamshire)	33.3% (Greater Manchester, but not Salford), 6.6% (North-West England, but not Greater Manchester)

Demographics between populations at the Royal Free differed from that at the Salford Care Organisation with a greater ethnic and language diversity seen at the Royal Free. The mean age of the participants at the time of them completing the survey was 43 years old. Disease activity assessed via the Bath Ankylosing Spondylitis Disease Activity Index (BASDAI) on the day of survey completion ranged from 0.4 to 9 out of 10. With regard to medication management, around 60% of the survey respondents were on biologic medications for their axial SpA at the time of the survey, with a greater percentage established on these medications in the Salford cohort compared to the Royal Free cohort. Across both rheumatology services, the average time using the mean calculation from the onset of symptoms to diagnosis was similar despite the differences in demographics, with Royal Free at 5.72 years and Salford Royal at 5.96 years. A recent publication has given a strong argument for the use of median rather than mean average, due to the tendency for mean average to be skewed, when considering axial SpA diagnostic delay [11]. When reviewing via median diagnostic delay, there was a notable difference with Royal Free at 6.09 years (IQR: 7.38) and Salford Royal at 4.27 years (IQR: 6.45).

**Figure 1:**
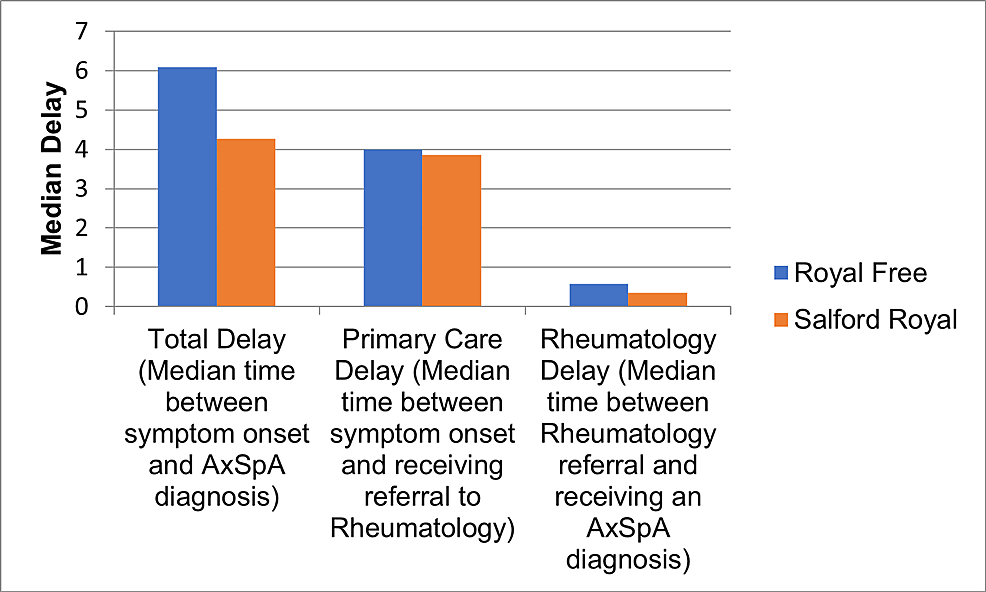
Median number of years of diagnostic delay at each part of the patient pathway to axial SpA diagnosis

Comparing the time from symptom onset to diagnosis for all participants by rheumatology service shows some disparity in primary care and secondary care delay (Figure 1). Of the total delay from onset to diagnosis, the time between first symptoms and a referral to rheumatology has a median of four years (IQR: 6.75) at Royal Free and 3.86 years (IQR: 6.36) at Salford Royal. The delay between the initial rheumatology consult in secondary care and the definitive diagnosis being made in-house at Salford Royal was 0.35 years (IQR: 0.33) and at the Royal Free 0.59 years (IQR: 2.00).

The survey also addressed disease-specific education at diagnosis and awareness of who to contact in the event of a flare; 80% of the survey respondents reported that they had received adequate axial SpA education at diagnosis, and 76% of the participants were aware of who to contact in the event of a flare.

**Figure 2:**
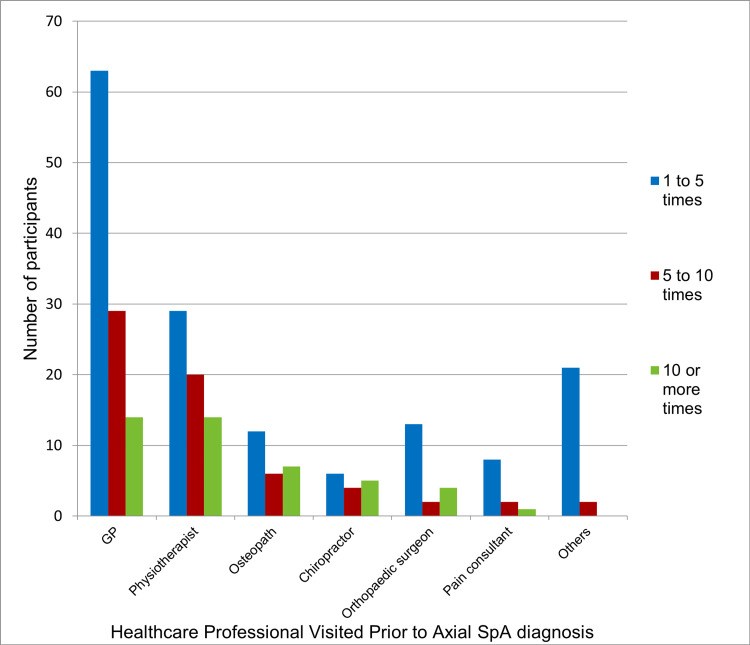
Number of patients visiting healthcare professionals with the number of times each healthcare professional was seen prior to axial SpA diagnosis The “Others” category includes visiting healthcare professionals from various specialities, including musculoskeletal, neurology, trauma, back pain, acupuncture, ophthalmologist, dermatology, gastroenterology and spinal.

Across the two sites, prior to diagnosis, 90% of the survey participants saw a GP, of which 63% saw a GP 1-5 times, 23% saw 5-10 times and 14% saw more than 10 times. Many participants also saw other HCPs (see Table 1) aside from the GP and rheumatologist prior to diagnosis: 32% saw one other HCPs, 18% two other HPCs, 9% three, 7% four and 2.7% five or more other HCPs prior to diagnosis. Figure 2 further outlines how many patients visited other HCPs and how many times they visited each healthcare professional prior to their referral to rheumatology services.

In comparing our data to previously published surveys, we were prompted to review if our delays to diagnosis were changing over time. Moran et al. (2016) broke down their responses as to when the diagnosis was made [12]. Table 2 displays our delay to diagnosis separated into timeframe sub-groups to allow comparison of the Lancaster data to that of our two rheumatology services. The spread of the Royal Free data does not allow a reliable comparison as more than 80% of the participants completing the survey at this site were diagnosed in the prior five years. Comparing the Salford Care Organisation data to the historic data from Lancaster does show a reasonable amount of similarity in patterns between the date of diagnosis sub-grouping.

**Table 2:** Delay to diagnosis data separated into sub-groups as to when the diagnosis was given The data for our two sites is compared to that of a previous study using the same timeframe sub-grouping. Mean (median) in years, N: number of participants in this time bracket

Date of a definitive diagnosis of axial SpA or AS	Royal Lancaster Infirmary	Salford Care Organisation, Northern Care Alliance NHS Foundation Trust	Royal Free NHS Foundation Trust
1962-1999	6.7 (7), N=10	4.7 (4.1), N=9	9 (9), N = 1
2000-2004	9.5 (7), N=13	7.9 (7.9), N=2	4.5 (4), N = 5
2005-2009	9.8 (8), N=15	5.4 (4.7), N=8	N=0
2010-2015	9.1 (7), N=7	8.1 (7.8), N=13	13.6 (5), N=3
2016-2021	No data here, paper published in 2016	4.9 (3.4), N=14	5.3 (4.1), N=46

## Discussion

The National Axial Spondyloarthritis Society (NASS) has an ongoing project ambitiously aiming to transform the average time to diagnosis throughout the NHS and other UK healthcare providers from 8 ½ years to just one year [13]. This Gold Standard Time to Diagnosis project will “deliver dramatic health and wellbeing improvements for those affected, enabling more patients to access appropriate treatment earlier and improve their health and well-being, thereby helping the NHS to become a global leader in axial SpA service delivery” [13].

Our survey results show a similarity to previously published data from other centres and also show a remarkable closeness across the two sites. This gives some confidence in the data and findings from this small, survey-based study. Overall, the delay to diagnosis from each of our sites is somewhat lower than previously published results; this may be due to the more recently diagnosed cohort tending to experience a shorter delay, with our data for those diagnosed in the 2016-2021 timeframe being significantly shorter in delay to diagnosis. This is a promising sign for the improvements in axial SpA diagnostic pathways in the past few years, but with this small sample size, definitive conclusions cannot be accepted, and instead, the overall pattern of no change in the delay to diagnosis will be further explored.

The observed lack of improvement in diagnostic delay over the 20-year period prior to 2016 found in our dataset concurs with other similar research [12] and larger datasets created from UK GP data in the Clinical Practice Research Datalink [14]. This lack of improvement has been suggested to be predominantly driven by delays in referral from primary care to rheumatology [14]. The article suggesting this primary care issue puts this down to both “The commonality of chronic back pain, and the complexity of AS, with diverse symptoms” [14]. These proposed issues in primary care are supported by research looking at the physiotherapists’ management of pre-diagnosis axial SpA. Steen et al. (2021), in a survey of UK practice, found that musculoskeletal physiotherapists may not be giving adequate consideration to axial SpA in back pain assessments [8]. Our data appear to agree with this, showing a majority of our participants having multiple physiotherapy appointments prior to any consideration of referral into rheumatology. The missed opportunity by physiotherapists is, however, still less of an issue from our dataset than that by GPs. A small piece of research on a subset of longer-delay axial SpA instances from our cohort at Salford Care Organisation did show some examples of how opportunities for earlier GP recognition of the need for rheumatology had been missed [7]. These delays are, to some extent, understandable given the variable and sometimes very subtle early presentation of axial SpA; however, they may well present as the cornerstone to improve diagnostic delay and do need further study.

The number of HCP appointments that a person with early axial SpA undertakes is a concern, as our study shows a majority of participants seeing their GP on multiple occasions prior to referral and one-third of participants seeing other HCPs in addition. These findings appear to agree with a 2019 survey of practice in the United States; Magrey et al. reported that 41% of their participants had seen multiple HCPs prior to a referral to rheumatology being undertaken [15]. The reasons suggested for HCP delay to refer include the perception of axial SpA as a male disease [16], the assumed requirement for raised inflammatory markers [8], the fact that axial SpA is a relatively uncommon cause of a common symptom (back pain) [16], the lack of pathognomic symptoms and signs of disease [13] and the lack of validated axial SpA diagnostic criteria [17].

Our survey data revealed 11 participants, out of 106 surveyed, who had seen one of the EAM secondary care specialities of dermatology, ophthalmology or gastrology prior to their referral into rheumatology. These one in 10 figures are lower than might be anticipated; the reported incidence of axial SpA is higher than this 10% in those with acute anterior uveitis (20%), inflammatory bowel disease (13%) and psoriasis (24%) [16].

Delay to diagnosis once under the care of rheumatology was significantly longer in the Royal Free dataset, and this is thought to correlate to the lack of a dedicated axial SpA service at this trust until very recently. The relatively quicker pathway from first rheumatology consult to definitive diagnosis at Salford Care Organisation may represent the role of the specialist axial SpA service or the increased disease awareness and diagnostic pathways this rheumatology focus has engendered. However, a review of cases and comparing the pathways at Salford Care Organisation do give an additional or alternative explanation, namely, that the referrals for queries of axial SpA are seen within the early inflammatory arthritis clinic and are triaged into this maximum three-week wait service as per National Early Inflammatory Arthritis Audit compliance requirements [18]; anecdotally, this incorporation of axial SpA into this rapid review new patient pathway is not a common practice in rheumatology departments.

Our survey data on patient education for those newly diagnosed with axial SpA is very much in line with other sources. The National Early Inflammatory Arthritis Audit in its most recent annual report demonstrated a very similar figure of 543/671 (81%) of respondents reporting diagnosis-specific education by three months post-diagnosis [18]. This is still some way off the 100% that we would like to achieve, and a review of post-diagnosis pathways is now being undertaken in each of our study sites. Similarly, our question on the survey with regard to an understanding of flare management and how to seek HCP support has identified areas for improvements locally in both sites.

Limitations

This has been a small survey with subsequent small power and also a survey with a cohort of convenience, sampling consecutive outpatient attendees. We cannot be sure if the participants accurately represent the full axial SpA cohorts at either of our two trusts. The majority of the participants recruited from the Royal Free site have a recent diagnosis, with 80% being diagnosed in the prior five years to the undertaking of the survey. Conversely, some of the participants at Salford Royal had such long-established diagnoses that accurate recall or contemporaneous medical records were unreliable. The more recent diagnosis bias of the Royal Free cohort of participants may make conclusions more difficult as these post-2010 diagnoses would have been made in a period where MRI scans were readily available, and the more recent Assessment of SpondyloArthritis international Society (ASAS) classification criteria [19] also allowed sooner diagnosis to be made. It is also noteworthy that the Royal Free cohort had a greater percentage of female participants than the Salford cohort: this may well represent the more even gender match in axial SpA diagnosis in recent years [16] but is certainly a limitation when comparing results from the two rheumatology service cohorts. The ethnic representation in the Royal Free cohort when compared to the Salford cohort accurately represents the local population diversity in each rheumatology service catchment area and hence was anticipated but is another variation across the two cohorts.

For all participants, survey-based research adds a risk of recall bias; we did attempt to minimise this by referring to electronic health records, but these were not available for all participants due to either their GPs’ lack of integration into the shared records or the time period the incidents happened; for instance, no electronic records from prior to 2004 for Salford Royal.

The signs and symptoms of early axial SpA are diverse and can be quite subtle; there is therefore a further limitation to this type of axial SpA delay to diagnosis survey. Our participants may have incorrectly attributed historical symptoms to their future axial SpA diagnosis; for instance, an episode of non-specific low back pain that would not fit the criteria of IBP could still be considered by some of our participants as their first axial SpA sign, hence inadvertently increasing their delay to diagnosis. In addition, the sparse nature of GP records means issues are most often recorded as “episodes of back pain” without any further details that might differentiate IBP from non-specific low back pain; although clearly, if the GP had suspected IBP, then referral would most likely have been made at this potentially earlier period.

## Conclusions

We have shown that delay in diagnosis remains a problem in the UK. Whilst our two sites have shown some improvement on previous published national delay data, we are each still a long way from the aspirational one-year maximum wait suggested by the NASS. Our patients are having too many “missed opportunities” for a referral to rheumatology; our survey, as one of the first to collect data on the number of HCPs seen, shows that multiple opportunities are missed by a wide variety of musculoskeletal HCPs. There are also indications that those secondary care services managing the EAMs seen in axial SpA could have been referred earlier in a few of our surveyed cases.

The two rheumatology services involved in this survey were prompted to undertake this study as a part of data collection for a quality improvement project. The projects at each site are now underway and have ongoing aims to reduce delays in diagnosis through a number of channels. Our findings imply that ongoing education and awareness events promoting the identification of inflammatory back pain signs and symptoms for both the general public and for community-based musculoskeletal HCPs would be a valuable use of time to significantly decrease delays in diagnosis in axial SpA.

## Appendices

Table 3 presents the 14-question survey used in the present study.

**Table 3:** The 14-question survey (Supplementary Material 1)

Diagnosis of Axial Spondyloarthritis Survey													
Thank you for taking the time to fill in our survey to help us improve our service. This survey will take you 2-3 minutes to complete. All compulsory questions are marked with a *.													
1	What is your gender?												
	Female	Male						Other				Prefer not to answer	
2	*What is your date of birth?												
3	*How would you describe your ethnicity?												
	White British				White Other					Black (African/Caribbean)			
	Asian (South Asia)				Asian (Far East)					Mixed/multiple			
	Other (please state)												
4	*What area do you live in?												
	(a) Salford version						(b) Royal Free version						
	Salford						Barnet						
	Greater Manchester						Watford						
	North-West						Hertfordshire						
	Other (please state)						Enfield						
							Other (please state)						
5	*What is your primary spoken language? (please state) free text												
6	*When did you first experience your Inflammatory back pain symptoms?												
	Month year												
7	*When did you first see your GP with regards to this problem?												
	Month year												
8	*When were you referred by a GP or another healthcare professional to a rheumatologist?												
	Month year												
9	*Which healthcare professionals did you see prior to your axial spondyloarthritis diagnosis and how many times did you see them?												
	GP		0			1-5			5-10				10+
	Physiotherapist		0			1-5			5-10				10+
	Osteopath		0			1-5			5-10				10+
	Chiropractor		0			1-5			5-10				10+
	Orthopaedic surgeon		0			1-5			5-10				10+
	Pain consultant		0			1-5			5-10				10+
	Other (please state)		0			1-5			5-10				10+
	Please state other professionals seen here												
10	*When were you formally diagnosed with axial spondyloarthritis/AS?												
	Month year												
11	*Were you provided with adequate patient information about your condition at diagnosis?												
	Yes			No							Unsure		
12	*Have you been given information about who to contact in a flare?												
	Yes			No							Unsure		
13	When did you start on biologics treatment?												
	Month year, N/A												
14	BASDAI score (office use only)												
